# PI3K/AKT pathway as a pivotal regulator of epithelial-mesenchymal transition in lung tumor cells

**DOI:** 10.1186/s12935-024-03357-7

**Published:** 2024-05-10

**Authors:** Meysam Moghbeli

**Affiliations:** Department of Medical Genetics and Molecular Medicine, School of Medicine, Mashhad University of Medical Sciences, Mashhad, Iran

**Keywords:** PI3K/AKT, EMT, Lung cancer, Metastasis

## Abstract

Lung cancer, as the leading cause of cancer related deaths, is one of the main global health challenges. Despite various progresses in diagnostic and therapeutic methods, there is still a high rate of mortality among lung cancer patients, which can be related to the lack of clinical symptoms to differentiate lung cancer from the other chronic respiratory disorders in the early tumor stages. Most lung cancer patients are identified in advanced and metastatic tumor stages, which is associated with a poor prognosis. Therefore, it is necessary to investigate the molecular mechanisms involved in lung tumor progression and metastasis in order to introduce early diagnostic markers as well as therapeutic targets. Epithelial-mesenchymal transition (EMT) is considered as one of the main cellular mechanisms involved in lung tumor metastasis, during which tumor cells gain the metastatic ability by acquiring mesenchymal characteristics. Since, majority of the oncogenic signaling pathways exert their role in tumor cell invasion by inducing the EMT process, in the present review we discussed the role of PI3K/AKT signaling pathway in regulation of EMT process during lung tumor metastasis. It has been reported that the PI3K/AKT acts as an inducer of EMT process through the activation of EMT-specific transcription factors in lung tumor cells. MicroRNAs also exerted their inhibitory effects during EMT process by inhibition of PI3K/AKT pathway. This review can be an effective step towards introducing the PI3K/AKT pathway as a suitable therapeutic target to inhibit the EMT process and tumor metastasis in lung cancer patients.

## Background

Lung cancer is the leading cause of cancer-related mortality globally. Non–small cell lung carcinoma (NSCLC) accounts 80% of lung cancers. There is a low survival rate in metastatic NSCLC patients because of the aggressive behavior of these tumors [1]. There are various therapeutic strategies such as surgery, chemotherapy, radiotherapy, and targeted therapy for NSCLC patients. However, there is still a low 5-years survival rate in NSCLC patients that is associated with late diagnosis in regional or distant metastasis [2, 3]. Regarding the deep location of lung tumors without any clear clinical symptoms in the early tumor stage, more than half of NSCLC patients are diagnosed in advanced stages with distant metastases [4, 5]. Therefore, it is required to elucidate the molecular mechanisms of lung tumor progression to improve early detection and prognosis. Epithelial mesenchymal transition (EMT) is a pivotal cellular process during tumor metastasis that is characterized with down-regulation of epithelial marker (E-cadherin), while up-regulation of mesenchymal markers and EMT-specific transcription factors (N-cadherin, Twist, Zeb1, Snail, and Slug) [6, 7]. During EMT process, tumor cells lose their cell-cell adhesions to obtain a mesenchymal feature with a high ability for invasion [8, 9]. E-cadherin (CDH1) down regulation as a hallmark of EMT is associated with Twist, Snail, Slug, and ZEB1 up regulations [10]. Various signaling pathways such as Mitogen-activated protein kinases (MAPK), phosphoinositide 3-kinase (PI3K)/protein kinase B (AKT), and Transforming growth factor-β (TGF-β) are also involved in regulation of EMT process [11–14]. Since, PI3K/AKT pathway has been reported to be activated in 90% of NSCLC cells; it can be introduced as a reliable target to inhibit the NSCLC progression [15]. Several inhibitors of PI3K/AKT/mTOR pathway have been introduced by preclinical and clinical trials in NSCLC patients [16]. GDC-0941 as a reversible inhibitor of PI3K was responsive to NSCLC cells with PIK3CA alteration and PTEN loss. A partial response was observed in a combination therapy by GDC-0941, paclitaxel, and carboplatin in NSCLC patients [17]. MK-2206 is an inhibitor of AKT that increases effect of RTK inhibitors and cytotoxic drugs in NSCLC cells [18]. Buparlisib (BKM120) is also an orally administered PI3K inhibitor that generates modest responses in lung cancer patients [19]. Temsirolimus inhibits NSCLC growth via reduced mTOR phosphorylation. It showed a partial response in NSCLC patients that lasted 12.7 months [20]. Therefore, in the present review we discussed the role of PI3K/AKT pathway in regulation of EMT process during lung tumor metastasis (Table 1).

**Table 1:** PI3K/AKT axis as a regulator of EMT process during lung tumor metastasis

Study	Year	Axis	Effect on the EMT	Samples	Clinical application
YIMINNIYAZE [24]	2023	EPHA3/PI3K/AKT	Induced	A549, H1299, H1975, and PC-9 cell lines	Diagnosis
LIU [25]	2020	miR-448/EPHA7/PI3K/AKT	Inhibited	51 NT* A549, H1299, H460, and SPC-A1 cell lines	Diagnosis and prognosis
LI [27]	2020	miR-147/BDNF/PI3K/AKT	Inhibited	79 patients A549 cell line	Diagnosis and prognosis
CAO [28]	2020	miR-1299/EGFR/PI3K/AKT	Inhibited	56 NT H1299, A549, H358, and H1975 cell lines	Diagnosis
LU [29]	2017	miR-92a/PTEN	Induced	50 NT A549, H358, SPC-A1, and H1299 cell lines	Diagnosis
SONG [33]	2021	PTPRN/PI3K/AKT	Induced	H1299 and A549 cell lines	Diagnosis and prognosis
YU [34]	2021	SNORA47/PI3K/AKT	Induced	A549 and NCI-H23 cell lines	Diagnosis
ZHAO [39]	2020	miR-625/RETN/PI3K/AKT/SNAIL	Inhibited	80 NT A549, H322, GLC-82, and H226 cell lines	Diagnosis and prognosis
JIA [40]	2018	miR-126/PI3K/AKT/SNAIL	Inhibited	SPC-A1 and LLC cell lines	Diagnosis
ZHOU [41]	2019	FAM83A/PI3K/AKT/SNAIL	Induced	101 patients PC-14, H661, A549, H827, PC-9, H1915, H2170, H460, and H1299 cell lines	Diagnosis and prognosis
WU [44]	2019	PAX6/ZEB2/PI3K/AKT	Induced	A549 and SPC-A1 cell lines	Diagnosis and prognosis
MA [48]	2019	miR-4458/AKT	Inhibited	A549, H1299, HCC827, PC-9, HBE, and 293T cell lines	Diagnosis
LIU [51]	2017	ING5/EGFR/PI3K/AKT	Inhibited	A549 and H1299 cell lines	Diagnosis
JIN [60]	2019	NETRIN1/PI3K/AKT	Induced	95 patients A549, H1299, H1975, SPC-A1, PC-9, and H522 cell lines	Diagnosis
XU [61]	2020	Circ-0018818/NID1/PI3K/AKT	Inhibited	30 NT A549, PC-9, H441, H1650, and 293T cell lines	Diagnosis
HU [64]	2021	CNTN1/PI3K/AKT	Induced	A549 cell line	Diagnosis
QIU [66]	2017	DAL-1/HSPA5/PI3K/AKT	Inhibited	A549, SPC-A1,HA579, H520, H460, and H1299 cell lines	Diagnosis
XUAN [69]	2019	miR-381/LMO/PI3K/AKT	Inhibited	54 NT A549, SPC-A1, H1299, and PC-9 cell lines	Diagnosis and prognosis
TANG [70]	2019	KIAA1199/PI3K/AKT	Induced	254 patients A549, H1299, H1975, and H1650 cell lines	Diagnosis
WANG [71]	2020	miR-874/AQP3/PI3K/AKT	Inhibited	49 NT A549 and H1299 cell lines	Diagnosis and prognosis
MA [74]	2019	ENKUR/PI3K/AKT	Inhibited	515T and 59 N A549, H322, PC-9, SPC-A1, and GLC-82 cell lines	Diagnosis
WANG [80]	2018	ELF3/PI3K/AKT	Induced	85T and 22 N SPC-A1 and A549 cell lines	Diagnosis and prognosis
LI [82]	2023	ZNF687/PI3K/AKT/GSK3β/SNAIL	Induced	98T and 82 N A549, PC-9, HCC827, and H1975 cell lines	Diagnosis and prognosis
LIN [85]	2021	ARHGAP10/PI3K/AKT/GSK3β	Inhibited	66 NT A549, H1299, H1975, and SKMES-1 cell lines	Diagnosis
KUANG [88]	2020	RNF8/PI3K/AKT/SLUG	Induced	1100 patients H1299, H1395, Calu-1, and A549 cell lines	Diagnosis and prognosis
LIU [90]	2017	TRIM22/PI3K/AKT/GSK3β/β-CATENIN	Induced	126 patients H460, A549, H358, LK2, H1299, and H3255 cell lines	Diagnosis and prognosis
JEON [94]	2017	PELI1/AKT/GSK3β	Induced	A549, CALU-3, CALU-6, H322, H358, H1650, H441, H460, H1299, H1264, PC-9, H827, H1833, H1838, H1975, H820, and H4006 cell lines	Diagnosis
LEE [97]	2017	APBB1/IGF1R/AKT/GSK3β	Induced	A549 and H460 cell lines	Diagnosis
YUAN [99]	2020	miR-410/PTEN/PI3K/AKT/Mtor	Induced	62 NT A549, H1299, PC-9, and SPC-A1 cell lines	Diagnosis
KHENDELWAL [100]	2021	miR-320a/PI3K/AKT/mTOR	Inhibited	80 patients A549 cell line	Diagnosis and prognosis
CHEN [104]	2016	miR-206/C-MET/PI3K/AKT/mTOR	Inhibited	34 NT A549 and H1299 cell lines	Diagnosis
MOU [106]	2016	miR-485/FLOT2/PI3K/AKT/mTOR	Inhibited	25 NT A549,H1650, H332, and SPC-A1 cell lines	Diagnosis and prognosis
CHEN [113]	2016	miR-206/HGF/C-MET/PI3K/AKT/mTOR	Inhibited	35 NT A549, 95D, 95 C, and 801 C cell lines	Diagnosis
ZHAO [116]	2021	HRH3/PI3K/AKT/mTOR	Induced	H460, A549, H1703, PC-9, and H1975 cell lines	Diagnosis
PENG [120]	2023	GPX2/PI3K/AKT/mTOR	Induced	293 patients H520, H358, H1299, H460, and A549 cell lines	Diagnosis and prognosis

* Tumor (T) tissues, Normal (N) margins

### PI3K/AKT axis

**Fig. 1:**
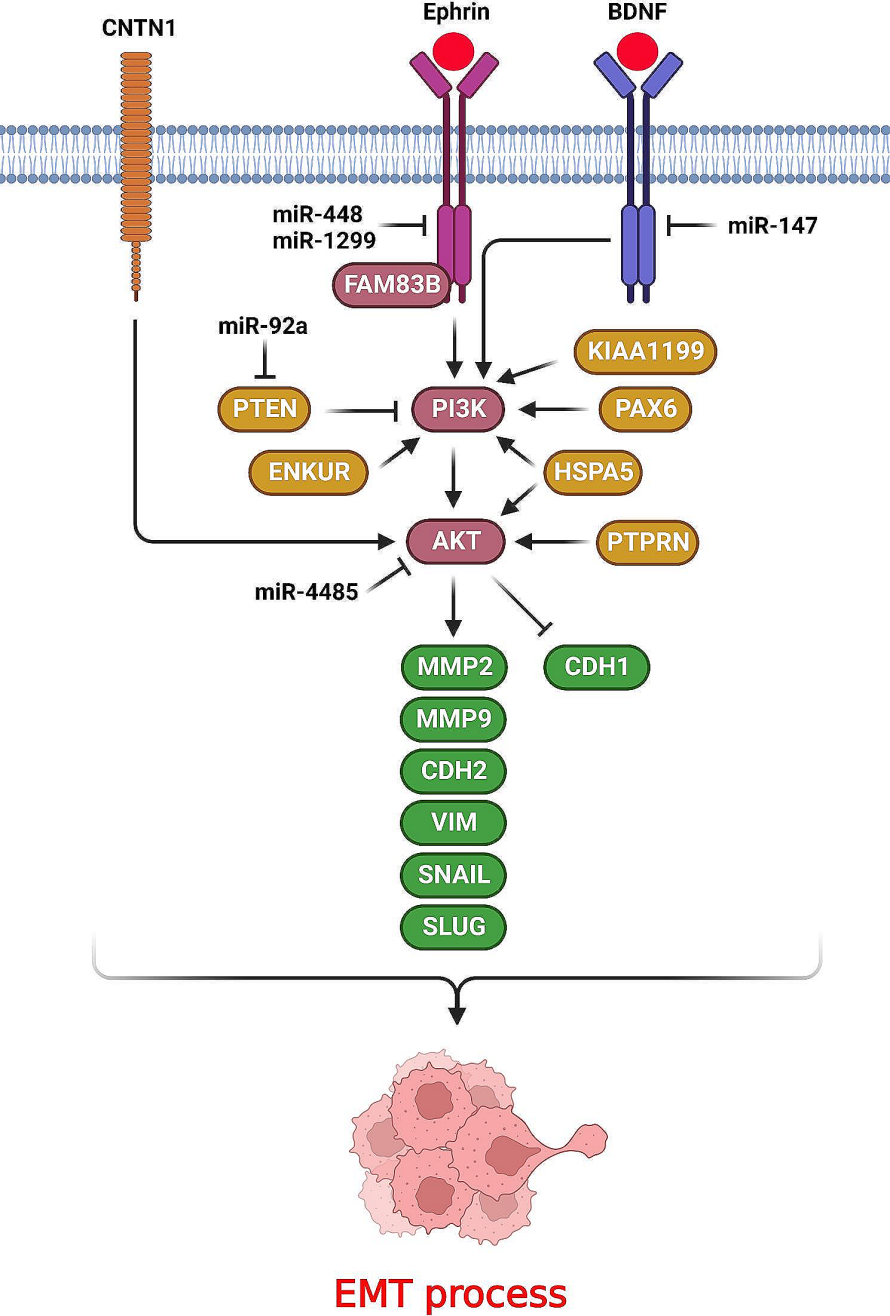
Role of PI3K/AKT axis in regulation of EMT process during lung tumor metastasis. (Created with *BioRender.com*)

Receptor tyrosine kinases (RTKs) have a fundamental role in regulation of cell proliferation, metabolism, migration, and apoptosis through the PI3K/AKT pathway [21]. It has been shown that PI3K/AKT pathway has a key role in regulation of EMT process during lung tumor metastasis (Fig. 1). Ephrin (Eph) is considered as the largest RTK subfamily that can be activated by Ephrin ligands to regulate cell adhesion and proliferation [22, 23]. EphrinA3 inhibition reduced the LUAD cell proliferation and migration. It promoted PI3K/Akt to up regulate the CCND1. It also regulates the EMT process by MMP2/9 up regulations, which are the key factors during lung adenocarcinoma (LUAD) metastasis [24]. There was significant miR-448 down regulation in NSCLC tissues that was associated with poor prognosis. MiR-448 inhibited PI3K/Akt pathway and EMT process via EPHA7 targeting in NSCLC cells [25]. Brain Derived Neurotrophic Factor (BDNF) is an activator of the tropomyosin-related tyrosine kinase (Trk) receptors to promote MAPK and PI3K signaling pathways. PI3K/AKT can be promoted by BDNF/TRKB and p75NTR axes [26]. There was miR-147 down regulation in NSCLC tissues that was correlated with poor prognosis, lymph node invasion, and tumor stage. MiR-147 inhibited the EMT process by Vimentin (VIM) and CDH2 down regulations while CDH1 up regulation in NSCLC. MiR-147 also inhibited PI3K/AKT pathway via p-PI3K and p-AKT down regulations in NSCLC cells [27]. EGFR is a well-known RTK that promotes cell growth and metabolism via PI3K/AKT and MAPK pathways. There was miR-1299 down regulation in NSCLC tissues compared with normal margins. MiR-1299 reduced NSCLC cell migration and EMT process via EGFR targeting [28]. Phosphatase and tensin homolog (PTEN) is a negative regulator of PI3K/AKT pathway that has mainly a tumor suppressor function in tumor cells. MiR-92a increased NSCLC cell invasion and EMT process through PTEN targeting [29]. Tumor microenvironment (TME) induces the immune escape as a hallmark of tumor progression [30]. There is a continuous correlation between the immune cells of microenvironment and tumor cells during tumor initiation to metastasis [31]. Protein Tyrosine Phosphatase Receptor Type N (PTPRN) is involved in insulin secretion of pancreatic islet β-cells [32]. There was PTPRN up regulation in LUAD tissues that was contributed with poor prognosis and metastasis. PTPRN increased LUAD cell metastasis through the regulation of PI3K/AKT pathway. It up regulated the VIM, CDH2, and p-AKT in LUAD cells [33]. Small nucleolar RNAs (snoRNAs) are non-coding RNAs that are involved in chemical modifications of rRNAs, tRNAs, and snRNAs. It has been reported that Small Nucleolar RNA, H/ACA Box 47 (SNORA47) inhibition reduced NSCLC progression and EMT process through PI3K/AKT pathway. SNORA47 down regulated CDH1 while up regulated CDH2 in NSCLC cells [34].

EMT can be regulated by Snail and Twist transcription factors [35, 36]. AKT as the main effector of PI3K up regulates the Snail/Twist transcription factors to down regulate CDH1. Resistin is an inflammo-regulatory protein that is involved in tumor progression [37]. It induces the tumor cell proliferation through the promotion of PI3K/AKT pathway [38]. There was an inverse association between the levels of Resistin and miR-625 expressions that was significantly correlated with lymph node invasion and tumor stage in NSCLC patients. Resistin increased NSCLC progression and EMT process through PI3K/AKT/Snail axis. MiR-625 inhibited NSCLC cell migration and EMT by Resistin targeting [39]. MiR-126 reduced lung cancer cells invasion through targeting the PI3K/AKT/Snail axis [40]. Family With Sequence Similarity 83 Member A (FAM83A) functions in Epidermal Growth Factor Receptor (EGFR) pathway by promotion of the PI3K/AKT/TOR axis. There was significant FAM83A up regulation in NSCLC tissues which was associated with poor prognosis. FAM83A induced NSCLC cell invasion by PI3K/ATK/Snail axis and EMT promotion [41].

Paired-box 6 (PAX6) is a developmental transcription factor that is involved in embryogenesis [42, 43]. There was significant PAX6 up regulation that was correlated with poor prognosis in NSCLC patients. It induced NSCLC cell migration via ZEB2 up regulation that reduced the levels of CDH1 expression through PI3K/AKT pathway. PAX6 up regulated the p-AKT, p-PI3K, CDH2, vimentin, while down regulated CDH1 [44]. High Mobility Group AT-Hook 1 (HMGA1) is a regulator of chromatin remodeling by binding to A/T-rich regions [45]. It has key roles in regulation of cell proliferation, invasion, and EMT process [46, 47]. It has been reported that miR-4458 inhibition up regulated p-AKT. miR-4458 reduced NSCLC cell migration and EMT via HMGA1 targeting [48]. Inhibitor of Growth (ING) protein family includes ING1-5 members that are involved in cell proliferation, apoptosis, and chromatin remodeling [49]. ING5 interacts with p300 and p53 via a zinc finger domain to promote apoptosis [50]. ING5 inhibition induced lung tumor cell invasion through promotion of the EGFR/PI3K/AKT mediated EMT process. ING5 had inhibitory role on EGFR/PI3K/AKT axis by p-AKT down regulation [51]. Sirtuin-1 (SIRT1) is a conserved histone deacetylase which has pivotal roles in epigenetic regulation through histones and non-histone modifications [52]. B7H3 (CD276) suppresses tumor associated T cell activation [53], while induces tumor cell invasion and drug resistance [54]. It was shown that B7H3 promoted the NSCLC cell invasion and EMT process. SIRT1 regulated the B7H3mediated EMT. Reciprocally, B7H3 also modulated SIRT1 through PI3K/AKT axis in NSCLC cells [55].

Hypoxia as a hallmark of fast-growing solid tumors has a pivotal role in metastasis that results in poor prognosis [56]. Since, Hypoxia promotes EMT in lung tumor cells, it is required to assess the molecular mechanisms of hypoxia mediated EMT to overcome the poor prognosis. Netrin-1 is a cell-secreted soluble protein that has key roles in tissue development and tumor cell migration [57, 58]. It promoted the PI3K/AKT mediated EMT via interaction with FAK. Therefore, Netrin-1 induces cell migration through activation of FAK/PI3K/AKT axis [59]. Hypoxia mediated Netrin-1 inhibition down regulated p-AKT that reduced NSCLC cell migration. Netrin-1 induced hypoxia-mediated EMT via PI3K/AKT pathway in NSCLC cells [60]. Nidogen 1 (NID1) is a sulfated glycoprotein associated with laminin that is involved in cellular interaction with extracellular matrix. Circ_0018818 inhibition reduced NSCLC tumor progression by miR-767-3p sponging that activated the NID1/PI3K/Akt/EMT axis [61]. Contactin-1 (CNTN1) is a neuronal adhesion protein involved in tumor progression [62, 63]. CNTN1 inhibition increased gefitinib sensitivity while inhibited EMT process through PI3K/AKT inactivation and cytoskeletal rearrangement in lung adenocarcinoma cells. CNTN1 inhibition down regulated the VIM and CDH2 while up regulated CDH1 [64].

Heat Shock Protein Family A Member 5 (HSPA5) is a member of the HSP70 protein family that is involved in regulation of EMT process and tumor metastasis [3, 65]. It was observed that DAL-1 suppressed EMT process and NSCLC cell proliferation via HSPA5 down regulation. DAL-1 mediated HSPA5 inhibition down regulated p-PI3K, p-Akt, and p-Mdm2 while up regulated p53 to attenuate EMT via suppressing the PI3K/AKT/Mdm2/p53 axis [66]. LIM-only protein 3 (LMO3) as a regulator of p53 is involved in cell growth and invasion [67, 68]. There was miR-381 down regulation in lung adenocarcinoma tissues that was correlated with poor prognosis. MiR-381 reduced lung adenocarcinoma cell proliferation and migration by LMO targeting and regulation of PI3K/Akt pathway and EMT process [69]. KIAA1199 as an endoplasmic reticulum (ER) protein has key roles in tumor invasion by Ca^2+^ release into the cytoplasm that activates protein kinase C to facilitate cell migration. It mediates hyaluronic acid (HA) depolymerization to regulate endocytosis. It regulates EMT by collaboration with HSPA5/BIP in a Ca^2+^ and PKC-related pathway. There was significant KIAA1199 up regulation in NSCLC tissues in comparison with normal controls. KIAA1199 promoted the EMT process during NSCLC progression and metastasis. KIAA1199 increased NSCLC invasion and EMT process via PI3K-Akt activation [70].

Aquaporins (AQPs) are a group of membrane channels that facilitate water transportation to regulate osmotic gradient. There was miR-874 down regulation in NSCLC tissues that was associated with poor prognosis. MiR-874 suppressed NSCLC cell invasion and EMT process by AQP3 targeting via regulation of PI3K/AKT axis. AQP3 activated the PI3K/AKT pathway via PI3K and AKT phosphorylations. Therefore, miR-874 suppressed EMT process via AQP3 targeting and subsequent inhibition of PI3K/AKT in NSCLC [71]. Enkurin (ENKUR) is considered as a Calmodulin (CaM)-binding protein that links the signal proteins with TRPC channels [72]. It also binds to the p85 subunit of PI3K [73]. ENKUR inhibition resulted in CDH1 down regulation while VIM and CDH2 up regulations in lung tumor cells. ENKUR also significantly down regulated PI3K and reduced p-Akt levels [74].

### PI3K/AKT/GSK3β axis

**Fig. 2:**
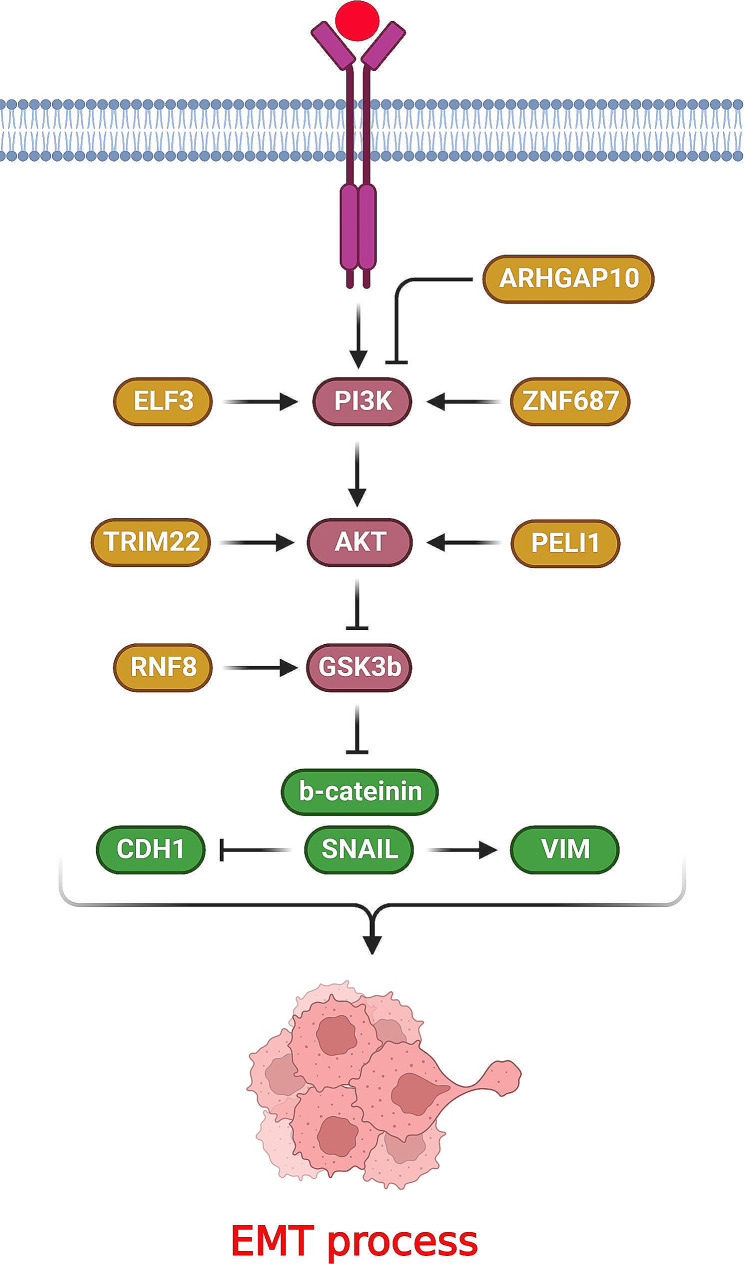
Role of PI3K/AKT/GSK3β axis in regulation of EMT process during lung tumor metastasis. (Created with *BioRender.com*)

Glycogen Synthase Kinase 3 Beta (GSK-3β) is a downstream target of PI3K/AKT and Extracellular Signal-Regulated Kinase (ERK) signaling pathways that can be inhibited by AKT or ERK [75]. Both these signaling pathways phosphorylate and inhibit the GSK-3β, that results in Snail and Slug up-regulation and EMT induction [76, 77]. It has been shown that PI3K/AKT/GSK3β axis has a key role in regulation of EMT process during lung tumor metastasis (Fig. 2). E74 Like ETS Transcription Factor 3 (ELF3) has key roles in tumor progression and embryogenesis [78, 79]. There was ELF3 up regulation in NSCLC that induced cell proliferation and invasion via activation of PI3K/Akt pathway. ELF3 up regulation was correlated with distant metastasis and clinical stages in NSCLC patients. There was also a negative association between the levels of ELF3 and survival rate in NSCLC patients. ELF3 inhibition reduced NSCLC cell growth by Cyclin D1 (CCND1), E2F Transcription Factor 1 (E2F1), and c-Myc down regulations. ELF3 induced EMT via CDH2, vimentin, Slug, and snail up regulations while CDH1 down regulation. ELF3 silencing reduced the levels of p-PI3K, p-GSK-3β and p-Akt expressions. Therefore, ELF3 increased NSCLC cell proliferation and invasion by PI3K/AKT activation and its downstream EMT related targets [80]. Zinc finger protein 687 (ZNF687) is a C2H2 zinc finger protein that has key roles in transcriptional regulation via binding to the ZNF592 and ZNF532 complex. ZNF687 is involved in epigenetic modulation and also transcriptional inhibition in DNA damaged regions [81]. ZNF687 up regulation was observed in LUAD tissues that were associated with poor prognosis. ZNF687 promoted G1/S phase progression by CDK2/4/6 and CCND1 up regulations while p27, p53, and p21 down regulations. ZNF687 inhibition reduced the levels of CDH2, VIM, MMP2, MMP9, and Snail expressions while up regulated CDH1, suggesting the regulatory role of ZNF687 on LUAD invasion and EMT process. ZNF687 inhibition also decreased the p-AKT, p-PDK1, and pGSK-3β levels. Therefore, ZNF687 was suggested as an EMT modulator via regulation of PI3K/AKT/GSK-3β/Snail axis that affected the LUAD cell metastasis [82]. Rho GTPase activating protein 10 (ARHGAP10) is involved in regulation of cell migration, cytoskeletal organization, and EMT process [83, 84]. There was significant ARHGAP10 down regulation in NSCLC tissues. ARHGAP10 reduced EMT process by CDH1 up regulation while CDH2, snail, and VIM down regulations. ARHGAP10 also reduced the levels of components of PI3K/Akt/GSK3β axis that reduced EMT in lung tumor cells [85].

Ring Finger Protein 8 (RNF8) as an ubiquitin E3 ligase has pivotal roles in regulation of cell proliferation, spermatogenesis, and apoptosis [86]. It induces the EMT by GSK-3β inhibition that results in accumulation of β-catenin and subsequent tumor metastasis [87]. RNF8 induced lung tumorigenesis through stabilization of Slug and PI3K/AKT signaling. There was RNF8 up regulation in lung tumor tissues that was conversely associated with patient’s survival [88]. Tripartite Motif Containing 22 (TRIM22) functions as a transcriptional regulator and E3 ubiquitin ligase [89]. There was significant TRIM22 up regulation in lung tumor tissues that was associated with poor prognosis. TRIM22 promoted the EMT process via Snail up regulation. It also increased the p-AKT levels. TRIM22 down regulated the CDH1 through snail following the AKT activation. Therefore, TRIM22 promoted EMT process via activation of PI3K/AKT/GSK3β/β-catenin axis in NSCLC cells [90]. Cancer stem cells (CSCs) are a small subpopulation of tumor cells that are involved in tumor recurrence via their self-renewal ability and drug resistance. EMT promotes tumor metastasis by generation of chemo resistant cancer stem cells [8, 91]. Pellino-1 as an E3 ubiquitin ligase is involved in immune response via regulation of T-cell receptor signaling and B and T cells activations [92, 93]. It also stabilizes the Snail and Slug through K63-mediated ubiquitination that induces the EMT process. Pellino-1 induced lung tumor cell proliferation by Akt activation that stabilized Slug and Snail. It also inhibited the GSK3β in lung tumor cells [94]. Amyloid Beta Precursor Protein Binding Family B Member 1 (APBB1) as an adaptor protein has critical role in cellular response toward genotoxic stress [95]. Insulin Like Growth Factor 1 (IGF1) binding to IGF1R activates insulin receptor substrates (IRS) to promote cell growth and migration [96]. APBB1 regulates EMT and radio resistance by activation of IGF1R/AKT/GSK3b axis in NSCLC cells [97].

### PI3K/AKT/mTOR axis

**Fig. 3:**
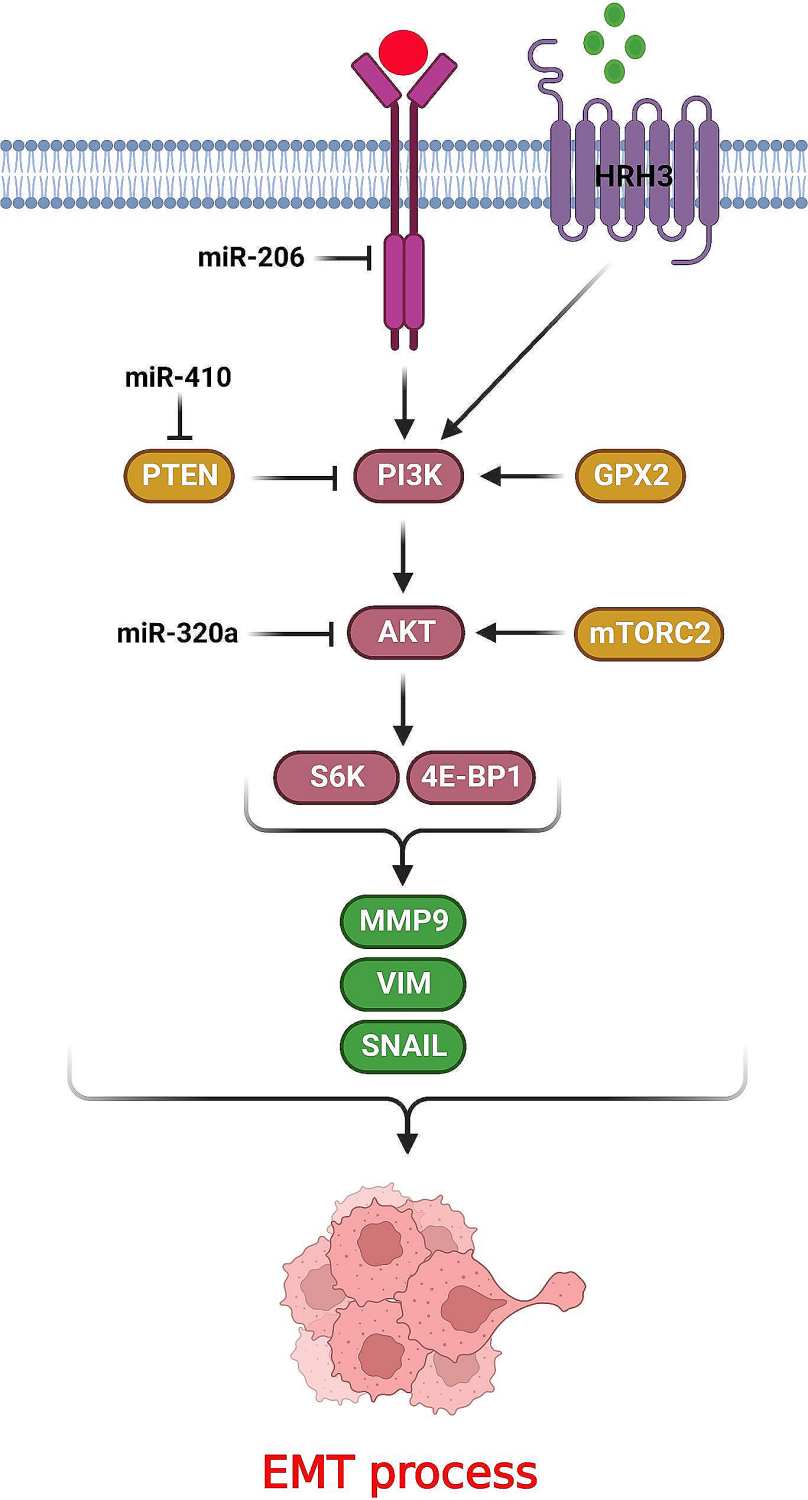
Role of PI3K/AKT/mTOR axis in regulation of EMT process during lung tumor metastasis. (Created with *BioRender.com*)

Mammalian target of rapamycin (mTOR) is a serine/threonine kinase that is one of the main effectors in PI3K/Akt pathway. mTOR refers to mTORC1 and mTORC2 complexes with different functions. mTORC1 promotes protein synthesis, cell metabolism, and growth by regulation of S6K1 and 4EBP1, while mTORC2 suppresses the Cyclin D1/E proteolysis via Akt activation [98]. Therefore, deregulation of PI3K/AKT/mTOR axis can be associated with neoplastic transformation [21]. It has been shown that PI3K/AKT/mTOR axis has a key role in regulation of EMT process during lung tumor metastasis (Fig. 3). MiR-410 promoted the EMT process and radio resistance by targeting the PTEN/PI3K/mTOR axis in NSCLC cells. MiR-410 up regulated the phosphorylated Akt and mTOR [99]. There was significant miR-320a down regulation in NSCLC samples that was correlated with TNM stage and poor prognosis. MiR-320a regulated the NSCLC progression via AKT3 targeting in PI3K/AKT/mTOR axis. MiR-320a inhibition up regulated CCND1, Matrix Metallopeptidase 9 (MMP9), Bcl-2, and β-catenin that increased cell proliferation and invasion in NSCLC [100]. Cisplatin (DDP) is one of the chemotherapeutic agents that are frequently used in lung cancer due to high efficiency and easy administration. However, there is a high rate of cisplatin resistance among patients [101]. It was observed that EMT process is involved in drug resistance of tumor cells [102, 103]. MiR-206 reduced EMT process and CDDP resistance via MET targeting that inhibited PI3K/AKT/mTOR axis in lung adenocarcinoma cells. EMT gene profile was significantly associated with MDR1 up regulation and CDDP resistance [104]. Flotillin 2 (FLOT2) is a caveolae-associated protein that is involved in vesicular trafficking and tumor progression [105]. There was miR-485 down regulation in lung adenocarcinoma tissues that was inversely associated with metastatic potential. MiR-485 inhibited the EMT process by FLOT2 targeting in lung adenocarcinoma cells. MiR-485 inhibited AKT and mTOR in lung adenocarcinoma that was reversed by FLOT2. Therefore, miR-485 promoted the PI3K/ AKT/mTOR axis by FLOT2 down regulation [106].

Angiogenesis has a key role in tumor progression and metastasis through preparing the required nutrients and oxygen for the tumor cells [107]. Angiogenic factors promote the endothelial cell proliferation to form the new vessels [108]. Both angiogenesis and EMT process can be stimulated by Vascular Endothelial Growth Factor (VEGF), Fibroblast Growth Factor (FGF), and Platelet Derived Growth Factor (PDGF) growth factors [109]. Hepatocyte Growth Factor (HGF) promotes metastatic potential of the tumor cells to spread in blood circulation via activation of the c-Met pathway [110, 111]. It is considered as an angiogenic cytokine that modifies the microenvironment via c-Met activation to facilitate tumor progression [112]. MiR-206 reduced HGF-mediated EMT and angiogenesis in lung cancer by c-Met targeting that resulted in suppression of PI3k/Akt/mTOR axis. MiR-206 also inhibited lung tumor growth and angiogenesis in vivo that introduced miR-206 as an efficient therapeutic target in lung cancer [113]. Histamine has a significant role in regulation of tumor-associated processes that exerts its role by binding to G protein-coupled receptors (GPCRs) including H1-4 histamine receptors. Histamine receptor H3 (Hrh3) inhibition reduces the tumor cell proliferation while promotes caspase-mediated apoptosis [114]. Hrh3 activates PI3K/AKT and MAPK signaling pathways to exert pathophysiological functions [115]. Hrh3 inhibition reduced NSCLC cell proliferation and metastasis via suppression of EMT process that is related to inhibition of PI3K/AKT/mTOR pathway [116]. Glutathione peroxidase 2 (GPX2) has a critical role in protection of cells toward the oxidative damages by hydrogen peroxide and fatty acid hydroperoxides reductions [117–119]. It has been shown that GPX2 up regulation was correlated with poor survival of NSCLC patients with lymph node invasion and advanced TNM stage. GPX2 up regulated the Snail and VIM, while down regulated CDH1 that finally increased NSCLC cell invasion. GPX2 inhibition reduced the levels of p-PI3K, p-AKT, and p-mTOR in NSCLC cells [120].

## Conclusions

A wide range of oncogenic signaling pathways can induce tumor cell invasion by promotion of the EMT process. In this study, we discussed the role of PI3K/AKT pathway in regulation of the EMT process during lung tumor metastasis. It has been shown that the PI3K/AKT pathway acts as an inducer of EMT process during lung tumor metastasis. Many tumor suppressors and miRNAs also exert their inhibitory effects on lung tumor metastasis and EMT through PI3K/AKT inhibition. This review can be an effective step in introducing the PI3K/AKT pathway as a suitable therapeutic target to inhibit the EMT process and tumor cell invasion in lung cancer patients. It has been shown that ncRNAs have a key role in regulation of the EMT process through PI3K/AKT pathway. Considering the inhibitory effect of miRNAs on the PI3K/AKT pathway as an inducer of the EMT process, miRNAs can be used as the reliable therapeutic targets via the miRNA mimics strategy. On the other hand, due to the inhibitory effects of lncRNAs and circRNAs on miRNAs, they can be also considered as the therapeutic targets to inhibit the PI3K/AKT mediated EMT process in the early stages of tumor metastasis. However, there is still not any clinical report about the application of ncRNAs to inhibit the EMT process through the PI3K/AKT pathway. Indeed, more animal studies and clinical trials are needed to use ncRNAs to inhibit PI3K/AKT mediated EMT process in lung cancer patients.

## Data Availability

The datasets used and/or analyzed during the current study are available from the corresponding author on reasonable request.

## Abbreviations

AQPs: Aquaporins
BDNF: Brain Derived Neurotrophic Factor
CaM: Calmodulin
CSCs: Cancer stem cells
DDP: Cisplatin
CNTN1: Contactin-1
CCND1: Cyclin D1
E2F1: E2F Transcription Factor 1
ELF3: E74 Like ETS Transcription Factor 3
CDH1: E-cadherin
ENKUR: Enkurin
EMT: Epithelial-mesenchymal transition
ERK: Extracellular Signal-Regulated Kinase
FAM83A: Family With Sequence Similarity 83 Member A
FGF: Fibroblast Growth Factor
FLOT2: Flotillin 2
GPCRs: G protein-coupled receptors
GPX2: Glutathione peroxidase 2
GSK-3β: Glycogen Synthase Kinase 3 Beta
HSPA5: Heat Shock Protein Family A Member 5
HGF: Hepatocyte Growth Factor
HMGA1: High Mobility Group AT-Hook 1
Hrh3: Histamine receptor H3
ING: Inhibitor of Growth
IRS: Insulin receptor substrates
LUAD: Lung adenocarcinoma
mTOR: Mammalian target of rapamycin
MMP9: Matrix Metallopeptidase 9
MAPK: Mitogen-activated protein kinases
NID1: Nidogen 1
NSCLC: Non–small cell lung carcinoma
PAX6: Paired-box 6
PTEN: Phosphatase and tensin homolog
AKT: Phosphoinositide 3-kinase (PI3K)/protein kinase B
PDGF: Platelet Derived Growth Factor
PTPRN: Protein Tyrosine Phosphatase Receptor Type N
RTKs: Receptor tyrosine kinases
ARHGAP10: Rho GTPase activating protein 10
RNF8: Ring Finger Protein 8
SIRT1: Sirtuin-1
SNORA47: Small Nucleolar RNA, H/ACA Box 47
snoRNAs: Small nucleolar RNAs
TGF-β: Transforming growth factor-β
TRIM22: Tripartite Motif Containing 22
Trk: Tropomyosin-related tyrosine kinase
TME: Tumor microenvironment
VEGF: Vascular Endothelial Growth Factor
VIM: Vimentin
ZNF687: Zinc finger protein 687

## References

[1] Sung WJ, Kim H, Park KK (2016). The biological role of epithelial-mesenchymal transition in lung cancer (review). Oncol Rep.

[2] Avelino CU, Cardoso RM, Aguiar SS, Silva MJ (2015). Assessment of quality of life in patients with advanced non-small cell lung carcinoma treated with a combination of carboplatin and paclitaxel. J Bras Pneumol.

[3] Chen HA, Chang YW, Tseng CF, Chiu CF, Hong CC, Wang W (2015). E1A-mediated inhibition of HSPA5 suppresses cell migration and invasion in triple-negative breast cancer. Ann Surg Oncol.

[4] Fischer C, Leithner K, Wohlkoenig C, Quehenberger F, Bertsch A, Olschewski A (2015). Panobinostat reduces hypoxia-induced cisplatin resistance of non-small cell lung carcinoma cells via HIF-1alpha destabilization. Mol Cancer.

[5] Maharati A, Zanguei AS, Khalili-Tanha G, Moghbeli M (2022). MicroRNAs as the critical regulators of tyrosine kinase inhibitors resistance in lung tumor cells. Cell Communication Signaling: CCS.

[6] Puisieux A, Brabletz T, Caramel J (2014). Oncogenic roles of EMT-inducing transcription factors. Nat Cell Biol.

[7] Mahmoudian RA, Akhlaghipour I, Lotfi M, Shahidsales S, Moghbeli M (2023). Circular RNAs as the pivotal regulators of epithelial-mesenchymal transition in gastrointestinal tumor cells. Pathol Res Pract.

[8] Thiery JP, Acloque H, Huang RY, Nieto MA (2009). Epithelial-mesenchymal transitions in development and disease. Cell.

[9] Hamidi AA, Khalili-Tanha G, Nasrpour Navaei Z, Moghbeli M (2022). Long non-coding RNAs as the critical regulators of epithelial mesenchymal transition in colorectal tumor cells: an overview. Cancer Cell Int.

[10] Cano A, Perez-Moreno MA, Rodrigo I, Locascio A, Blanco MJ, del Barrio MG (2000). The transcription factor snail controls epithelial-mesenchymal transitions by repressing E-cadherin expression. Nat Cell Biol.

[11] Willis BC, Borok Z (2007). TGF-beta-induced EMT: mechanisms and implications for fibrotic lung disease. Am J Physiol Lung Cell Mol Physiol.

[12] Moghbeli M, Makhdoumi Y, Soltani Delgosha M, Aarabi A, Dadkhah E, Memar B (2019). ErbB1 and ErbB3 co-over expression as a prognostic factor in gastric cancer. Biol Res.

[13] Maharati A, Moghbeli M (2023). PI3K/AKT signaling pathway as a critical regulator of epithelial-mesenchymal transition in colorectal tumor cells. Cell Communication Signaling: CCS.

[14] Maharati A, Moghbeli M (2023). Long non-coding RNAs as the critical regulators of PI3K/AKT, TGF-beta, and MAPK signaling pathways during breast tumor progression. J Transl Med.

[15] Chen M, Du Y, Qui M, Wang M, Chen K, Huang Z (2013). Ophiopogonin B-induced autophagy in non-small cell lung cancer cells via inhibition of the PI3K/Akt signaling pathway. Oncol Rep.

[16] Alharbi KS, Shaikh MAJ, Almalki WH, Kazmi I, Al-Abbasi FA, Alzarea SI (2022). PI3K/Akt/mTOR pathways inhibitors with potential prospects in Non-small-cell Lung Cancer. J Environ Pathol Toxicol Oncology: Official Organ Int Soc Environ Toxicol Cancer.

[17] Cheng H, Shcherba M, Pendurti G, Liang Y, Piperdi B, Perez-Soler R (2014). Targeting the PI3K/AKT/mTOR pathway: potential for lung cancer treatment. Lung Cancer Manag.

[18] Rao G, Pierobon M, Kim IK, Hsu WH, Deng J, Moon YW (2017). Inhibition of AKT1 signaling promotes invasion and metastasis of non-small cell lung cancer cells with K-RAS or EGFR mutations. Sci Rep.

[19] Massacesi C, Di Tomaso E, Urban P, Germa C, Quadt C, Trandafir L (2016). PI3K inhibitors as new cancer therapeutics: implications for clinical trial design. Onco Targets Ther.

[20] Porta C, Paglino C, Mosca A (2014). Targeting PI3K/Akt/mTOR signaling in Cancer. Front Oncol.

[21] Navaei ZN, Khalili-Tanha G, Zangouei AS, Abbaszadegan MR, Moghbeli M (2021). PI3K/AKT signaling pathway as a critical regulator of cisplatin response in tumor cells. Oncol Res.

[22] Himanen JP, Nikolov DB (2003). Eph receptors and ephrins. Int J Biochem Cell Biol.

[23] Kania A, Klein R (2016). Mechanisms of ephrin-eph signalling in development, physiology and disease. Nat Rev Mol Cell Biol.

[24] Yiminniyaze R, Zhang X, Zhu N, Wang J, Li C, Wumaier G (2023). EphrinA3 is a key regulator of malignant behaviors and a potential prognostic factor in lung adenocarcinoma. Cancer Med.

[25] Liu HY, Chang J, Li GD, Zhang ZH, Tian J, Mu YS (2020). MicroRNA-448/EPHA7 axis regulates cell proliferation, invasion and migration via regulation of PI3K/AKT signaling pathway and epithelial-to-mesenchymal transition in non-small cell lung cancer. Eur Rev Med Pharmacol Sci.

[26] Sandhya VK, Raju R, Verma R, Advani J, Sharma R, Radhakrishnan A (2013). A network map of BDNF/TRKB and BDNF/p75NTR signaling system. J Cell Commun Signal.

[27] Li F, Wang X, Yang L (2020). MicroRNA-147 targets BDNF to inhibit cell proliferation, migration and invasion in non-small cell lung cancer. Oncol Lett.

[28] Cao S, Li L, Li J, Zhao H (2020). MiR-1299 impedes the progression of Non-small-cell Lung Cancer through EGFR/PI3K/AKT signaling pathway. Onco Targets Ther.

[29] Lu C, Shan Z, Hong J, Yang L (2017). MicroRNA-92a promotes epithelial-mesenchymal transition through activation of PTEN/PI3K/AKT signaling pathway in non-small cell lung cancer metastasis. Int J Oncol.

[30] Hanahan D, Weinberg RA (2011). Hallmarks of cancer: the next generation. Cell.

[31] Perez-Ruiz E, Melero I, Kopecka J, Sarmento-Ribeiro AB, Garcia-Aranda M, De Las Rivas J (2020). Cancer immunotherapy resistance based on immune checkpoints inhibitors: targets, biomarkers, and remedies. Drug Resist Updat.

[32] Toledo PL, Torkko JM, Muller A, Wegbrod C, Sonmez A, Solimena M (2019). ICA512 RESP18 homology domain is a protein-condensing factor and insulin fibrillation inhibitor. J Biol Chem.

[33] Song X, Jiao X, Yan H, Yu L, Jiang L, Zhang M (2021). Overexpression of PTPRN promotes metastasis of lung adenocarcinoma and suppresses NK Cell cytotoxicity. Front Cell Dev Biol.

[34] Yu H, Tian L, Yang L, Liu S, Wang S, Gong J (2021). Knockdown of SNORA47 inhibits the tumorigenesis of NSCLC via Mediation of PI3K/Akt signaling pathway. Front Oncol.

[35] Lamouille S, Xu J, Derynck R (2014). Molecular mechanisms of epithelial-mesenchymal transition. Nat Rev Mol Cell Biol.

[36] Abbaszadegan MR, Taghehchian N, Li L, Aarabi A, Moghbeli M (2018). Contribution of KCTD12 to esophageal squamous cell carcinoma. BMC Cancer.

[37] Ibrahim DM, Shaaban ESE, Fouad TA (2020). Circulating Resistin is Associated with plasma glucagon-like Peptide-1 in cirrhotic patients with Hepatitis C Virus Genotype-4 infection. Endocr Res.

[38] Kim HJ, Lee YS, Won EH, Chang IH, Kim TH, Park ES (2011). Expression of resistin in the prostate and its stimulatory effect on prostate cancer cell proliferation. BJU Int.

[39] Zhao Y, Zheng R, Ning D, Xie F (2020). MiR-625 inhibits Tumor Cell Invasion, Migration and EMT by negatively regulating the expression of Resistin in Non-small Cell Lung. Cancer Manag Res.

[40] Jia Z, Zhang Y, Xu Q, Guo W, Guo A (2018). miR-126 suppresses epithelial-to-mesenchymal transition and metastasis by targeting PI3K/AKT/Snail signaling of lung cancer cells. Oncol Lett.

[41] Zhou F, Geng J, Xu S, Meng Q, Chen K, Liu F (2019). FAM83A signaling induces epithelial-mesenchymal transition by the PI3K/AKT/Snail pathway in NSCLC. Aging.

[42] Zhang J, Lu JP, Suter DM, Krause KH, Fini ME, Chen B (2010). Isoform- and dose-sensitive feedback interactions between paired box 6 gene and delta-catenin in cell differentiation and death. Exp Cell Res.

[43] Zhang X, Yang X, Wang J, Liang T, Gu Y, Yang D (2015). Down-regulation of PAX6 by promoter methylation is associated with poor prognosis in non small cell lung cancer. Int J Clin Exp Pathol.

[44] Wu DM, Zhang T, Liu YB, Deng SH, Han R, Liu T (2019). The PAX6-ZEB2 axis promotes metastasis and cisplatin resistance in non-small cell lung cancer through PI3K/AKT signaling. Cell Death Dis.

[45] Fusco A, Fedele M (2007). Roles of HMGA proteins in cancer. Nat Rev Cancer.

[46] Zhang Z, Wang Q, Chen F, Liu J (2015). Elevated expression of HMGA1 correlates with the malignant status and prognosis of non-small cell lung cancer. Tumour Biol.

[47] Zhong J, Liu C, Zhang QH, Chen L, Shen YY, Chen YJ (2017). TGF-beta1 induces HMGA1 expression: the role of HMGA1 in thyroid cancer proliferation and invasion. Int J Oncol.

[48] Ma Y, Li X, Chen S, Du B, Li Y (2019). MicroRNA-4458 suppresses migration and epithelial-mesenchymal transition via targeting HMGA1 in non-small-cell lung cancer cells. Cancer Manag Res.

[49] Qi L, Zhang Y (2014). Truncation of inhibitor of growth family protein 5 effectively induces senescence, but not apoptosis in human tongue squamous cell carcinoma cell line. Tumour Biol.

[50] Shiseki M, Nagashima M, Pedeux RM, Kitahama-Shiseki M, Miura K, Okamura S (2003). p29ING4 and p28ING5 bind to p53 and p300, and enhance p53 activity. Cancer Res.

[51] Liu XL, Zhang XT, Meng J, Zhang HF, Zhao Y, Li C (2017). ING5 knockdown enhances migration and invasion of lung cancer cells by inducing EMT via EGFR/PI3K/Akt and IL-6/STAT3 signaling pathways. Oncotarget.

[52] Sauve AA, Wolberger C, Schramm VL, Boeke JD (2006). The biochemistry of sirtuins. Annu Rev Biochem.

[53] Cai D, Li J, Liu D, Hong S, Qiao Q, Sun Q (2020). Tumor-expressed B7-H3 mediates the inhibition of antitumor T-cell functions in ovarian cancer insensitive to PD-1 blockade therapy. Cell Mol Immunol.

[54] Yu TT, Zhang T, Lu X, Wang RZ (2018). B7-H3 promotes metastasis, proliferation, and epithelial-mesenchymal transition in lung adenocarcinoma. Onco Targets Ther.

[55] Liao H, Ding M, Zhou N, Yang Y, Chen L. B7–H3 promotes the epithelial–mesenchymal transition of NSCLC by targeting SIRT1 through the PI3K/AKT pathway. Mol Med Rep. 2022;25(3). 10.3892/mmr.2022.12595 PMC8778653 35029291

[56] Ye LY, Chen W, Bai XL, Xu XY, Zhang Q, Xia XF (2016). Hypoxia-Induced epithelial-to-mesenchymal transition in Hepatocellular Carcinoma induces an immunosuppressive Tumor Microenvironment to Promote Metastasis. Cancer Res.

[57] Dominici C, Moreno-Bravo JA, Puiggros SR, Rappeneau Q, Rama N, Vieugue P (2017). Floor-plate-derived netrin-1 is dispensable for commissural axon guidance. Nature.

[58] Akino T, Han X, Nakayama H, McNeish B, Zurakowski D, Mammoto A (2014). Netrin-1 promotes medulloblastoma cell invasiveness and angiogenesis, and demonstrates elevated expression in tumor tissue and urine of patients with pediatric medulloblastoma. Cancer Res.

[59] Zhang Y, Wang B, Chen X, Li W, Dong P (2017). AGO2 involves the malignant phenotypes and FAK/PI3K/AKT signaling pathway in hypopharyngeal-derived FaDu cells. Oncotarget.

[60] Jin X, Luan H, Chai H, Yan L, Zhang J, Wang Q (2019). Netrin–1 interference potentiates epithelial–to–mesenchymal transition through the PI3K/AKT pathway under the hypoxic microenvironment conditions of non–small cell lung cancer. Int J Oncol.

[61] Xu X, Zhou X, Gao C, Cui Y (2020). Hsa_circ_0018818 knockdown suppresses tumorigenesis in non-small cell lung cancer by sponging miR-767-3p. Aging.

[62] Wang B, Yang X, Zhao T, Du H, Wang T, Zhong S (2020). Upregulation of contactin-1 expression promotes prostate cancer progression. Oncol Lett.

[63] Yan J, Wong N, Hung C, Chen WX, Tang D (2013). Contactin-1 reduces E-cadherin expression via activating AKT in lung cancer. PLoS ONE.

[64] Hu CS, Huang JH, Yang DL, Xu C, Xu ZG, Tan HB (2021). Lentivirus-mediated silencing of CNTN1 enhances gefitinib sensitivity by reversing epithelial-mesenchymal transition in lung adenocarcinoma A549 cells. Oncol Lett.

[65] Yu T, Guo Z, Fan H, Song J, Liu Y, Gao Z (2016). Cancer-associated fibroblasts promote non-small cell lung cancer cell invasion by upregulation of glucose-regulated protein 78 (GRP78) expression in an integrated bionic microfluidic device. Oncotarget.

[66] Qiu X, Guan X, Liu W, Zhang Y (2017). DAL-1 attenuates epithelial to mesenchymal transition and metastasis by suppressing HSPA5 expression in non-small cell lung cancer. Oncol Rep.

[67] Qiu YS, Jiang NN, Zhou Y, Yu KY, Gong HY, Liao GJ (2018). LMO3 promotes gastric cancer cell invasion and proliferation through Akt-mTOR and Akt-GSK3beta signaling. Int J Mol Med.

[68] Larsen S, Yokochi T, Isogai E, Nakamura Y, Ozaki T, Nakagawara A (2010). LMO3 interacts with p53 and inhibits its transcriptional activity. Biochem Biophys Res Commun.

[69] Xuan YW, Liao M, Zhai WL, Peng LJ, Tang Y (2019). MicroRNA-381 inhibits lung adenocarcinoma cell biological progression by directly targeting LMO3 through regulation of the PI3K/Akt signaling pathway and epithelial-to-mesenchymal transition. Eur Rev Med Pharmacol Sci.

[70] Tang Z, Ding Y, Shen Q, Zhang C, Li J, Nazar M (2019). KIAA1199 promotes invasion and migration in non-small-cell lung cancer (NSCLC) via PI3K-Akt mediated EMT. J Mol Med (Berl).

[71] Wang S, Wu Y, Yang S, Liu X, Lu Y, Liu F (2020). miR-874 directly targets AQP3 to inhibit cell proliferation, mobility and EMT in non-small cell lung cancer. Thorac Cancer.

[72] Sutton KA, Jungnickel MK, Wang Y, Cullen K, Lambert S, Florman HM (2004). Enkurin is a novel calmodulin and TRPC channel binding protein in sperm. Dev Biol.

[73] Clark JD, Gebhart GF, Gonder JC, Keeling ME, Kohn DF (1997). Special Report: the 1996 guide for the Care and Use of Laboratory animals. ILAR J.

[74] Ma Q, Lu Y, Lin J, Gu Y (2019). ENKUR acts as a tumor suppressor in lung adenocarcinoma cells through PI3K/Akt and MAPK/ERK signaling pathways. J Cancer.

[75] Saiprasad G, Chitra P, Manikandan R, Sudhandiran G (2014). Hesperidin induces apoptosis and triggers autophagic markers through inhibition of Aurora-A mediated phosphoinositide-3-kinase/Akt/mammalian target of rapamycin and glycogen synthase kinase-3 beta signalling cascades in experimental colon carcinogenesis. Eur J Cancer.

[76] Zhao J, Ou B, Han D, Wang P, Zong Y, Zhu C (2017). Tumor-derived CXCL5 promotes human colorectal cancer metastasis through activation of the ERK/Elk-1/Snail and AKT/GSK3beta/beta-catenin pathways. Mol Cancer.

[77] Zhou SL, Zhou ZJ, Hu ZQ, Li X, Huang XW, Wang Z (2015). CXCR2/CXCL5 axis contributes to epithelial-mesenchymal transition of HCC cells through activating PI3K/Akt/GSK-3beta/Snail signaling. Cancer Lett.

[78] Neve RM, Parmar H, Amend C, Chen C, Rizzino A, Benz CC (2006). Identification of an epithelial-specific enhancer regulating ESX expression. Gene.

[79] Oliver JR, Kushwah R, Hu J (2012). Multiple roles of the epithelium-specific ETS transcription factor, ESE-1, in development and disease. Lab Invest.

[80] Wang H, Yu Z, Huo S, Chen Z, Ou Z, Mai J (2018). Overexpression of ELF3 facilitates cell growth and metastasis through PI3K/Akt and ERK signaling pathways in non-small cell lung cancer. Int J Biochem Cell Biol.

[81] Malovannaya A, Lanz RB, Jung SY, Bulynko Y, Le NT, Chan DW (2011). Analysis of the human endogenous coregulator complexome. Cell.

[82] Li M, Liu Z, Hou Z, Wang X, Shi H, Li Y (2023). Oncogenic zinc finger protein ZNF687 accelerates lung adenocarcinoma cell proliferation and tumor progression by activating the PI3K/AKT signaling pathway. Thorac Cancer.

[83] Barcellos KS, Bigarella CL, Wagner MV, Vieira KP, Lazarini M, Langford PR (2013). ARHGAP21 protein, a new partner of alpha-tubulin involved in cell-cell adhesion formation and essential for epithelial-mesenchymal transition. J Biol Chem.

[84] Basseres DS, Tizzei EV, Duarte AA, Costa FF, Saad ST (2002). ARHGAP10, a novel human gene coding for a potentially cytoskeletal Rho-GTPase activating protein. Biochem Biophys Res Commun.

[85] Lin LL, Yang F, Zhang DH, Hu C, Yang S, Chen XQ (2021). ARHGAP10 inhibits the epithelial-mesenchymal transition of non-small cell lung cancer by inactivating PI3K/Akt/GSK3beta signaling pathway. Cancer Cell Int.

[86] Lu LY, Wu J, Ye L, Gavrilina GB, Saunders TL, Yu X (2010). RNF8-dependent histone modifications regulate nucleosome removal during spermatogenesis. Dev Cell.

[87] Kuang J, Li L, Guo L, Su Y, Wang Y, Xu Y (2016). RNF8 promotes epithelial-mesenchymal transition of breast cancer cells. J Exp Clin Cancer Res.

[88] Kuang J, Min L, Liu C, Chen S, Gao C, Ma J (2020). RNF8 promotes epithelial-mesenchymal transition in Lung Cancer cells via stabilization of slug. Mol Cancer Res.

[89] Duan Z, Gao B, Xu W, Xiong S (2008). Identification of TRIM22 as a RING finger E3 ubiquitin ligase. Biochem Biophys Res Commun.

[90] Liu L, Zhou XM, Yang FF, Miao Y, Yin Y, Hu XJ (2017). TRIM22 confers poor prognosis and promotes epithelial-mesenchymal transition through regulation of AKT/GSK3beta/beta-catenin signaling in non-small cell lung cancer. Oncotarget.

[91] Moghbeli M, Mosannen Mozaffari H, Memar B, Forghanifard MM, Gholamin M, Abbaszadegan MR (2019). Role of MAML1 in targeted therapy against the esophageal cancer stem cells. J Transl Med.

[92] Chang M, Jin W, Chang JH, Xiao Y, Brittain GC, Yu J (2011). The ubiquitin ligase Peli1 negatively regulates T cell activation and prevents autoimmunity. Nat Immunol.

[93] Chang M, Jin W, Sun SC (2009). Peli1 facilitates TRIF-dependent toll-like receptor signaling and proinflammatory cytokine production. Nat Immunol.

[94] Jeon YK, Kim CK, Hwang KR, Park HY, Koh J, Chung DH (2017). Pellino-1 promotes lung carcinogenesis via the stabilization of slug and snail through K63-mediated polyubiquitination. Cell Death Differ.

[95] Saeki K, Nose Y, Hirao N, Takasawa R, Tanuma S (2011). Amyloid precursor protein binding protein Fe65 is cleaved by caspases during DNA damage-induced apoptosis. Biol Pharm Bull.

[96] Haisa M (2013). The type 1 insulin-like growth factor receptor signalling system and targeted tyrosine kinase inhibition in cancer. J Int Med Res.

[97] Lee JH, Kim JY, Kim SY, Choi SI, Kim KC, Cho EW (2017). APBB1 reinforces cancer stem cell and epithelial-to-mesenchymal transition by regulating the IGF1R signaling pathway in non-small-cell lung cancer cells. Biochem Biophys Res Commun.

[98] Paplomata E, O’Regan R (2014). The PI3K/AKT/mTOR pathway in breast cancer: targets, trials and biomarkers. Ther Adv Med Oncol.

[99] Yuan Y, Liao H, Pu Q, Ke X, Hu X, Ma Y (2020). miR-410 induces both epithelial-mesenchymal transition and radioresistance through activation of the PI3K/mTOR pathway in non-small cell lung cancer. Signal Transduct Target Ther.

[100] Khandelwal A, Sharma U, Barwal TS, Seam RK, Gupta M, Rana MK (2021). Circulating miR-320a acts as a tumor suppressor and prognostic factor in non-small cell Lung Cancer. Front Oncol.

[101] Tan XL, Moyer AM, Fridley BL, Schaid DJ, Niu N, Batzler AJ (2011). Genetic variation predicting cisplatin cytotoxicity associated with overall survival in lung cancer patients receiving platinum-based chemotherapy. Clin Cancer Res.

[102] Kajiyama H, Shibata K, Terauchi M, Yamashita M, Ino K, Nawa A (2007). Chemoresistance to paclitaxel induces epithelial-mesenchymal transition and enhances metastatic potential for epithelial ovarian carcinoma cells. Int J Oncol.

[103] Sun L, Yao Y, Liu B, Lin Z, Lin L, Yang M (2012). MiR-200b and miR-15b regulate chemotherapy-induced epithelial-mesenchymal transition in human tongue cancer cells by targeting BMI1. Oncogene.

[104] Chen QY, Jiao DM, Wang J, Hu H, Tang X, Chen J (2016). miR-206 regulates cisplatin resistance and EMT in human lung adenocarcinoma cells partly by targeting MET. Oncotarget.

[105] Wang YL, Yao WJ, Guo L, Xi HF, Li SY, Wang ZM (2015). Expression of flotillin-2 in human non-small cell lung cancer and its correlation with tumor progression and patient survival. Int J Clin Exp Pathol.

[106] Mou X, Liu S (2016). MiR-485 inhibits metastasis and EMT of lung adenocarcinoma by targeting Flot2. Biochem Biophys Res Commun.

[107] Fakhrejahani E, Toi M (2012). Tumor angiogenesis: pericytes and maturation are not to be ignored. J Oncol.

[108] Griffioen AW (2007). Therapeutic approaches of angiogenesis inhibition: are we tackling the problem at the right level?. Trends Cardiovasc Med.

[109] Kong W, He L, Richards EJ, Challa S, Xu CX, Permuth-Wey J (2014). Upregulation of miRNA-155 promotes tumour angiogenesis by targeting VHL and is associated with poor prognosis and triple-negative breast cancer. Oncogene.

[110] Gao D, Vahdat LT, Wong S, Chang JC, Mittal V (2012). Microenvironmental regulation of epithelial-mesenchymal transitions in cancer. Cancer Res.

[111] Gentile A, Trusolino L, Comoglio PM (2008). The Met tyrosine kinase receptor in development and cancer. Cancer Metastasis Rev.

[112] Meng F, Wu G (2012). The rejuvenated scenario of epithelial-mesenchymal transition (EMT) and cancer metastasis. Cancer Metastasis Rev.

[113] Chen QY, Jiao DM, Wu YQ, Chen J, Wang J, Tang XL (2016). MiR-206 inhibits HGF-induced epithelial-mesenchymal transition and angiogenesis in non-small cell lung cancer via c-Met /PI3k/Akt/mTOR pathway. Oncotarget.

[114] Tanaka S, Sakaguchi M, Yoneyama H, Usami Y, Harusawa S, Histamine (2016). H(3) receptor antagonist OUP-186 attenuates the proliferation of cultured human breast cancer cell lines. Biochem Biophys Res Commun.

[115] Bongers G, Bakker RA, Leurs R (2007). Molecular aspects of the histamine H3 receptor. Biochem Pharmacol.

[116] Zhao YY, Jia J, Zhang JJ, Xun YP, Xie SJ, Liang JF (2021). Inhibition of histamine receptor H3 suppresses the growth and metastasis of human non-small cell lung cancer cells via inhibiting PI3K/Akt/mTOR and MEK/ERK signaling pathways and blocking EMT. Acta Pharmacol Sin.

[117] Brigelius-Flohe R, Kipp A (2009). Glutathione peroxidases in different stages of carcinogenesis. Biochim Biophys Acta.

[118] Esworthy RS, Doroshow JH, Chu FF (2022). The beginning of GPX2 and 30 years later. Free Radic Biol Med.

[119] Wingler K, Muller C, Schmehl K, Florian S, Brigelius-Flohe R (2000). Gastrointestinal glutathione peroxidase prevents transport of lipid hydroperoxides in CaCo-2 cells. Gastroenterology.

[120] Peng F, Xu Q, Jing X, Chi X, Zhang Z, Meng X (2023). GPX2 promotes EMT and metastasis in non-small cell lung cancer by activating PI3K/AKT/mTOR/Snail signaling axis. FASEB Bioadv.

